# Navigating DNACPR decisions: a qualitative study of DNACPR factor variability and implementation among NHS Doctors

**DOI:** 10.1186/s12910-025-01286-2

**Published:** 2026-01-19

**Authors:** Silan Fidan, Simon Cohn

**Affiliations:** 1 Anaesthetics - Royal Free Hospital, Pond St, NW3 2QG London, UK; 2 London School of Hygiene and Tropical Medicine - Keppel St, WC1E 7HT London, UK

**Keywords:** DNACPR, CPR, Resuscitation, Futility, Frailty, Quality of life

## Abstract

**Background:**

The ongoing absence of a national Do Not Attempt Cardiopulmonary Resuscitation (DNACPR) policy means that there remains a lack of explicit guidance on how clinicians should select, measure and assess relevant factors when deciding whether to implement a DNACPR order. Although previous studies have raised this as a concern, this qualitative study explores the extent to which individual clinicians not only adopt personal strategies to assess patients, but also value having a degree of professional autonomy when making DNACPR decisions.

**Methods:**

Sixteen semi-structured interviews were conducted with National Health Service (NHS) clinicians via video calls. Participants were recruited via snowball sampling. Thematic analysis was conducted using an inductive approach. Initial codes were generated from the interview transcripts, which were then organised into overarching themes based on patterns and relationships identified across the coded data.

**Results:**

Four main groupings summarise the different considerations adopted by doctors when making DNACPR decisions: age, frailty, assessment of quality of life, and perceived outcome. However, whilst the same general areas for consideration were drawn on, the participating doctors nevertheless assessed them differently and often prioritised them in different ways. In the absence of explicit, standardised guidance for weighting these factors, decisions were often made on the basis of individual judgement and local convention.

**Conclusion:**

This study demonstrates that physician-level variation in DNACPR decision-making reflects the inherent tension clinicians experience when balancing individual patient needs with the absence of explicit, standardised guidance. The ability to make decisions on a case-by-case basis, with the freedom to weigh diverse factors in a nuanced way is an important aspect of clinical decision-making. However, this can lead to inadvertent bias if decisions are based on factors not necessarily supported by established evidence.

**Recommendations:**

Our findings highlight a clear need for a more robust and equitable framework for these critical conversations, whilst also enabling clinicians to be able to make case-by-case decisions. The development of a national DNAPR policy that integrates evidence-based data may help doctors select and weigh factors appropriately, and provide a framework for the initiation of DNACPR discussions. However, such a policy must nevertheless be sufficiently flexible to permit doctors the autonomy required to make nuanced decisions on the basis of a range of patient-specific contextual assessments. Our key recommendation, therefore, is that clinicians should be supported not only by improved procedures and processes, but also by confidence that their professional judgement is rooted in the most current clinical evidence.

**Supplementary Information:**

The online version contains supplementary material available at 10.1186/s12910-025-01286-2.

## Introduction

Although cardiopulmonary resuscitation (CPR) is a potentially life-preserving intervention given in situations of cardiac or respiratory arrest, the chances of survival to discharge following in-hospital resuscitation in the UK are approximately 20% [1]. Do not attempt cardiopulmonary resuscitation (DNACPR) orders are made by NHS doctors to withhold CPR for patients during their hospital admission on the basis that this low success rate and the risk of a traumatic and undignified death are not in the patient’s best interest [2]. The legal justification for DNACPR orders is rooted in the Human Rights Act 1998 [3] and the Mental Capacity Act 2005 [4].

The current practice of completing DNACPR orders across the NHS in England is based on three separate national frameworks: one provided by the General Medical Council (GMC) [2], one produced by the European Resuscitation Council (ERC) [5] and guidance issued jointly by the British Medical Association (BMA), the Resuscitation Council UK, and the Royal College of Nursing [6]. In addition, all UK NHS Trusts (local-level NHS organisations) are expected to publish their own guidelines [6]. Some local healthcare institutions actively involve patients and relatives by providing extensive information and facilitating discussion, whereas others adopt a more paternalistic role and tend to regard DNACPR orders as a technical concern, hence solely a medical determination.

Table 1 illustrates a few of the differences across current guidelines. A core issue with this is not that clinicians may vary how they make assessments on a case-by-case basis, but that the current lack of any singular, unambiguous policy risks *unintentional* variation if a doctor prioritises certain factors over others. Indeed, professional and personal life experiences may well mean clinicians draw on a range of social and cultural values that inadvertently perpetuate forms of unconscious bias [7]. Furthermore, despite all existing guidelines warning against making decisions solely according to crude measures such as age, comorbidity status, or the ambiguous category of frailty, in practice clinicians may resort to prioritising these rather than having sufficient time to assess a complex case holistically. A meta-analysis revealed that doctors often employ a cognitive model that overemphasises objective factors such as age compared to more complex assessments [8], while a survey using clinical case vignettes not only revealed inconsistencies between doctors’ predictions of survival, but also that DNACPR decisions were linked to their professional grades and specialties [9]. More recently, related concerns were voiced during the COVID-19 pandemic, when blanket DNACPR orders were hurriedly implemented in various care settings based solely on patient age and disability status, prompting high-profile inquiries into the legality of this response [10]. The underlying tension across all these concerns is that carefully considered DNACPR decisions often take time, but that when under pressure, clinicians may understandably draw solely on a limited number of apparently unambiguous and objective criteria.

**Table 1:** Extracts from current guidelines

European Resuscitation Council (ERC)	The value of an outcome to an individual will likely be specific to that person. Defining as a society, healthcare provider or even as a relative that a certain life no longer is worth living, especially when this becomes balanced against cost or societal interaction, should only be done with the greatest caution as it incorporates a great inherent risk of quickly crossing acceptable ethical boundaries. Individual autonomy gives individuals the right to decline a treatment but does not obligate a health system to provide a treatment that is either futile or not cost-effective. To make patient-centred decisions about the appropriateness of resuscitation requires clinicians and patients to have a shared understanding of how the patient defines a good outcome. The patient’s perspective on outcome may be influenced by factors such as age, religion, societal values, and personal experiences. This should inform decisions about treatments, such as CPR. Key challenges for clinicians are effective communication of uncertainty about the likely outcome if an individual has a cardiac arrest, and to ensure that their personal values and preferences do not influence the patient.
Guidance from the BMA, Resuscitation Council UK and Royal College of Nursing	Although the healthcare team may doubt whether the risks associated with CPR are justified by a very small chance of success, the individual who’s life is at stake may be willing to accept that chance. Realistic information must be provided sensitively to people about the nature of CPR, the chance of success in their specific circumstances and the likely risks, including the long-term neurological damage, if patients still ask that no DNACPR decision be made, this should usually be respected.
General Medical Council (GMC)	If a patient wishes to receive CPR and it is your considered judgement that CPR would not be clinically appropriate for the patient, you must sensitively explore their reasons for requesting it, their understanding of what it would involve, and their expectations about the likely outcome. As part of this, you should make sure that they have accurate information about the nature of and, for example, the length of survival and level of recovery that they might realistically expect if they were successfully resuscitated.

Although there has been considerable health service research examining DNACPR order implementation and the challenges faced by clinicians and healthcare organisations—including communication difficulties, policy variations, and barriers to appropriate discussions and decision-making [11], limited research exists exploring how individual clinicians prioritise and weigh different factors within their personal decision-making processes. Understanding these individual processes is particularly important given that they are likely to rely on personal, subjective judgements, such as assessing futility in light of resource limitations [12] or long term quality of life predictions. However, exploring decisions retrospectively is difficult because simply having a DNACPR order can have an adverse impact on the care patients receive, potentially increasing the risk of deterioration and cardiac arrest [11, 13, 14]. This may create a ‘looping effect’ where the original clinical decision appears validated by subsequent patient outcomes that may have been influenced by the order itself. The ongoing debate surrounding assisted dying in the UK as the Terminally Ill Adults (End of Life) Bill progresses through Parliament [15] lends particular urgency and relevance to understanding the variations in how medical professionals approach end-of-life decisions, including the critical area of DNACPR orders.

This study takes a qualitative approach to explore the main factors considered by clinicians when making DNACPR decisions. Its focus is not on establishing whether particular decisions can be deemed clinically appropriate, or on whether decisions have adverse clinical effects, but on exploring just how diverse the factors considered potentially relevant may be. This question has important implications, since different factors—with different values associated with them—may well not be considered commensurate or even have any form of equivalence. Drawing on a small sample of participants, this qualitative study does not seek generalisable findings but instead aims to obtain rich data illustrating the scope of issues clinicians consider, identifying where factors may conflict, and exploring individual strategies clinicians use to resolve these tensions. The study focuses on highlighting the inherent variation and subjectivity in these clinical decisions, rather than identifying specific patient characteristics subject to unconscious bias.

## Methods

Semi-structured in-depth interviews were conducted via video calls by the principal researcher (SF) with sixteen NHS doctors working at different NHS trusts across England. A semi-structured interview guide (provided in supplementary materials) was developed for this study to facilitate exploratory conversations with participants. The guide provided a framework of key topics and questions while allowing the flexibility to pursue emerging themes and insights as they arose during interviews. Participants were recruited via snowball sampling, and interviews took place between 2021 and 2024. Clinicians from NHS Wales were not actively recruited due to the different DNACPR policy framework (the “Sharing and Involving” policy) that operates in Wales [16]. Interviews lasted between 30 and 45 min. The interviews were recorded and transcribed via an automated transcription service. The transcripts were then manually checked and edited by the principal researcher to ensure accuracy before analysis. The transcripts were analysed using Braun and Clarke’s inductive thematic analysis approach [17]. Following familiarisation with the transcripts, codes were developed systematically by identifying features relevant to the research question. Codes were collated to search for themes based on patterns, relationships and overarching topics. The themes were reviewed to ensure codes formed coherent patterns and accurately represented the dataset. Themes were revised and refined throughout the data collection and analysis stages and were critically assessed by both authors to establish a consensus.

This study aims to use detailed qualitative data to reveal individual variation in the DNACPR decision-making process and examine whether this variation represents an inevitable tension between necessary clinical flexibility and the risk of unconscious bias. Importantly, the interviews were not about identifying whether some clinicians might make errors or be misguided but rather aimed to explore the presence and nature of variation in current practice. By employing maximum variation purposive sampling, we were able to recruit clinicians representing various specialties and hospitals. This intentional diversity was crucial for capturing a broad range of perspectives, experiences, and practices [18].

Verbal and written consent was obtained from each participant following explanation of the study aims and an opportunity to address any questions that were raised. Participants were reminded of their ability to stop interviews and end their participation in the study at any stage. All participants were anonymised and assigned a participant number for use in the analysis phase of the study, and details in the transcripts that might identify other individuals or institutions were redacted to establish confidentiality. Recordings were deleted following transcription. Table 2 outlines the grade and specialty of each participant. Formal ethical approval was granted by the host university, London School of Hygiene and Tropical Medicine (LSHTM), as well as confirmation of appropriate research ethics compliance obtained from the NHS Health Research Authority (HRA).

**Table 2:** Medical specialties and grades of the participants

Participant number	Medical speciality and grade
1	General Practitioner (GP) - Specialist Registrar
2	Acute Medicine - Specialist Registrar
3	GP
4	Emergency Medicine - Senior House Officer
5	Trauma and Orthopaedics -Core Surgical Trainee
6	Ophthalmology - Specialist Registrar
7	Acute Medicine - Foundation Year 3 Doctor
8	GP - Specialist Registrar
9	Anaesthetics and Intensive Care Medicine - Consultant
10	Cardiology - Specialist Registrar
11	Anaesthetics and Intensive Care Medicine - Consultant
12	Emergency Medicine - Specialist-Registrar
13	Medicine for the Elderly - Consultant
14	Medicine for the-Elderly - Consultant
15	Cardiology - Consultant
16	Medicine for the Elderly - Consultant

## Results

### Main thematic groupings

Four main groupings summarise the different considerations raised by participants when discussing which factors they may consider when making DNACPR decisions: assessments of quality of life, frailty, age, and outcome. The quotes in Tables 3, 4, 5 and 6 illustrate the nuanced and diverse cognitive processes underlying these considerations, highlighting the overlap and interconnected nature of how clinicians navigate between these factors in practice.

### Quality of life

Quality of life (QoL) emerged as a major concept that influences the DNACPR decisions of most of the participants in this study (*n* = 13). Of these, two-thirds said that they rely on their own personal assessments to make a general evaluation; some primarily based this on the patient’s current baseline situation, some based it on trying to predict what a patient’s quality of life might be following the return of spontaneous circulation (ROSC), whereas others tried to establish a general view on the basis of both the current and likely future QoL a patient will have. The indicators of poor baseline QoL mentioned were: reduced mobility, the presence of medical comorbidities, dependence on others for care, frequent hospital admissions, increased age, and inability to interact with one’s family. Poor QoL following ROSC was associated with physical and/or cognitive disability, dependence on others for care, admission to a care home, high burden of care on family members, return to an empty home, and inability to interact with family long-term.

The remaining third of the participants who referred to QoL in relation to making DNACPR decisions said that they also try to ascertain the patient’s own perspective on the matter; for example, whether they are satisfied with their current QoL or what an acceptable QoL might be for them following ROSC.

**Table 3:** Extracts from participants’ discussions of QoL

Participant 1	I think you do get a sense of whether people are fulfilled in their lives or whether they’re nearing the end of their lives. I have to be honest, and say, I probably do not ask people what a favourable outcome would be for them, because I’m so afraid of people going through needless resuscitation attempts. So their home environment, does it look like, you know, are they living in one room? Are they living in a micro environment? Are they mobile? Are they getting out to their garden?
Participant 2	Goal for me personally, would be, you want someone to be able to return to an acceptable standard of living for them personally, they define it.
Participant 3	For me, it is whether a patient will come off a ventilator or not. That is the main thing for me. I do not think my thought processes go beyond that. I very much try and put it in the hands of the patient to almost drive the conversation to get them to say yes.
Participant 4	We think about where we’re going to discharge the patient to, are we going to send them to a care home? Are they going to send it back to the family? Or are they going back to an empty house that they came from?
Participant 6	So if one’s more independent, I tend to associate that with a greater quality of life. Another thing is probably age, like a person who’s 50, or 60, arguably has a lot more responsibilities, a lot of things occurring in their life, whereas I guess someone who’s like 90, they have lived their life, lived a large part of their life.
Participant 7	I do notice that sometimes, if the quality of life beforehand is not good, sometimes that can kind of sway decision making.
Participant 9	If you get the impression from the patient, or from their next of kin, that they would not want to carry on living, even in the best possible realistic outcome from the condition, then you begin from there to discuss limitations in their care.
Participant 11	For me, successful CPR would be a patient who goes on to have a good neurological recovery at some point in the future and without significant physical or neurological disabilities that mean that they wish they hadn’t survived, or that their family wish that I hadn’t survived, you know, and where the burden of the limitations that they’re left with, and the burden of caring for that person on their family and their loved ones, is not so great as to mean that they wish that the person had died.

### Frailty

Eleven participants stated that the concept of frailty was an influential factor in making their DNACPR decisions. Five of these used the Clinical Frailty Scale (CFS) measurement system. However, there was some variation when this was adopted; some felt that the most relevant frailty score was a few weeks or months prior to admission, whereas others drew on a score at the time of trying to make a decision. Alternative frailty assessments included making less structured, case-by-case judgements about mobility, being able to carry out daily living activities, the degree to which a patient depended on others, and physical appearance during admission.

Importantly, the stated rationale for taking frailty into account in DNACPR decisions differed among participants. Reasons included the idea that it would help predict the likelihood of achieving ROSC, that it suggested the likelihood of withstanding intensive care unit (ICU) intervention following successful resuscitation, or that it served as a proxy or complementary method to evaluate quality of life either in the present or following ROSC.

**Table 4:** Extracts from participants’ discussions of frailty

Participant 1	I think that although obviously you should not discriminate against people who are immobile, I think it can be an indicator perhaps of things like frailty, and frailty being linked to DNACPR being less successful. Also frailty is their baseline kind of living circumstances- it’s really difficult to define, it sounds like I’m just discriminating against people who are immobile but I think what I’m trying to say is that it gives you the context of someone’s life.
Participant 5	We probably just make a very normative decision whether someone is frail or not frail…we never see them how they are at home, we always just see them when they’ve broken their hip, or they’ve got a chest infection and it’s a very acute thing, and they may look pale, or they may be bedridden, or things like that. So that’s what we would probably term as someone being thin, frail.
Participant 6	Like a ninety year old, who’s sort of living independently walking without any sort of aids, regardless of what capabilities they have, you automatically have an impression that they are somewhat well, whereas An eight year old, whose bed bound, who needs to be posted from bed to chair, you don’t really see them to be particularly healthy, and you probably think they have other comorbidities as well….and you think about a patient’s mobility status. So what is their quality of life currently? Are they sort of bed bound? Are they sort of living an active life?
Participant 8	Frailty is essentially having a poor post physiological reserve, or even cognitive reserve, so not necessarily age. However, But, but, you know, the poor physiological reserve or the frailty that can sometimes be associated with age…but not age.
Participant 13	Just because I know what a clinical frailty score is, doesn’t mean, I will pretend to know what that individual’s life is with a frailty score of six. So really, what I can give them is an understanding into the degree of organic damage that they might suffer and what it might mean for functioning.
Participant 15	Simple questions asking family about how they have been doing. I start with the last few weeks, a few months and then a year to make a really informed decision about it. Rather than score, because the score can be just a very rigid one and it can lead to misinterpretation. So it is more of a detailed analysis of mobility.

### Age

Overlapping with the concerns described above, patient age also emerged as a significant factor in nine of the interviews. Of these, most respondents referred to the physiological implications of increasing age, such as the presence of comorbidities or poor mobility, and therefore implicitly the likelihood of increasing frailty and reducing quality of life. However, in contrast to these considerations, age was also used more explicitly as a measure of remaining life expectancy and a vague assessment of a patient’s current responsibilities and achievements, suggesting that decisions sometimes involve judgements about the relative value of extending life for older versus younger patients. In addition, while some felt that increasing age was associated with a poor likelihood of successful rehabilitation, others focused on the idea that increasing age impacted the success of ROSC or the likelihood of leaving the ICU.

**Table 5:** Extracts from participants’ discussion of age

Participant 3	Age has never really come into it for me. I don’t think it should.
Participant 6	Like a person who’s 50, or 60, arguably has a lot more responsibilities, a lot of things occurring in their life, whereas I guess someone who’s like 90, they’ve lived their life, lived a large part of their life.
Participant 8	The older a patient is with, the more comorbidities they have, the less likely it is, the more likely the patient is going to have significant disability, even if CPR is successful…I guess, you’re thinking about, quality of life, in terms of you know that their baseline is probably worse than it was when they were 20 or 30 years old, but also that it is far more likely to take a significant decline if CPR is successful.
Participant 9	While you’re dealing with a finite resource in intensive care and medical support, and when you get beyond 85–90, and you’re seriously ill, the chances of a good outcome are so poor…and so I think age does play a part.
Participant 10	I suppose you start thinking about a DNACPR when people get over a certain age. But it shouldn’t be that big of a factor. If you had an infinite number of intensive care beds and like, sure, if every 85-year-old with COPD wanted to be intubated, then, I mean, it’d be fine, but there’s not an endless amount of money to do that. And so, you know, I think sometimes we’re a little bit dishonest about that. I think COVID brought that to light a little bit
Participant 11	So we know that the older you are, particularly once you get to sort of over 70/80 unfortunately, the reversibility is less and their capacity to recover also.
Participant 14	You can have two patients the same age, one of them sits at home all day and does nothing, but the other one is out doing lots of walks so their capacity to withstand a cardiac arrest, and to get better and get back to baseline is probably greater compared to that patient. The patient themselves, in discussing it with them, what are their views on everything? What do they think, obviously, not trying to say they have a choice sort of thing.

### Successful outcomes

When talking about what ‘successful’ CPR means, participants referred to a range of outcomes rather than any single indicator. Rather than any definitive set of criteria or order of priority, success was thought to be some kind of combination of the following: a patient returning to their baseline level of physical functioning, a good QoL as judged by the patient (or the clinician), minimal physical or neurological disability, a high degree of independence following ROSC, ROSC, or simply discharge from the hospital.

**Table 6:** Extracts from participants’ discussion of successful outcomes

Participant 3	I guess the outcome is to bring about, like, spontaneous circulation, I guess.
Participant 4	I’d say a successful outcome would be the quality of someone’s life when they get discharged from hospital post CPR.
Participant 5	Getting them back and getting them treated in some way, shape, or form.
Participant 6	Successful CPR would be one, in which you achieve ROSC and where the patient has a somewhat decent quality of life afterwards. And there’s not too much, you know, trauma or other sort of injuries caused by the CPR. I mean, even if it’s unsuccessful, if it’s the right patient to try it in, then I think it’s the right thing and technically, it was the right thing to do and it’s technically still a success in my mind, the fact that we tried it.
Participant 12	Are they likely to survive the discharge from hospital?
Participant 14	Obviously, you’re trying to do your best for a patient who you think would otherwise recover back to their baseline. So successfully means a quick resuscitation, and then you’re likely to get the patient back to their baseline and how they were- you know- before they came into hospital.

## Discussion

Significant variation was demonstrated in what factors participants considered when reflecting on how they made DNACPR decisions, as well as how these variables are then operationalised in practice. In addition, a range of indicators were used to assess each of these factors—ranging from established scoring systems to very personal strategies to make evaluations—introducing even further variability between participants. This study therefore reveals a fundamental conflict in DNACPR decision-making: the clinical necessity of individualised judgement exists alongside the risk of unconscious bias. Rather than simply documenting variation, our findings suggest that this variation reflects competing imperatives that may be impossible to fully reconcile.

Although other research similarly describes the potentially wide range of factors that clinicians draw on when making such decisions [19, 20], this study highlights the extent to which the conceptualisations of these factors often overlap for individual physicians. This suggests that the factors identified may often act as proxy measures for one another, or potentially for other ones that are not made explicit and fall outside the main themes we have identified. We therefore discuss the potential ambiguity of each of the main groupings below.

### Age

Despite conflicting evidence concerning the impact of age on survival to hospital discharge following CPR [8, 21], many participants talked about age as a predictor of outcomes following ROSC. This finding is consistent with a large international study of patients with renal disease, which revealed that the chance of having a DNACPR order increased by 16% for each ten-year increase in age; this effect persisted after adjusting for multiple medical and socioeconomic factors [22]. A similar result was reproduced in a multicentre blinded simulation study carried out in the UK [23].

Medical and legal bodies such as the Care Quality Commission (CQC) and BMA have warned against the use of age in determining CPR status, as it could lead to unlawful practices under the Equality Act 2010 [24, 25]. Given the degree of heterogeneity in the health and functional status of patients within age groups, and the lack of prospectively examined evidence on the quality of outcomes following CPR, drawing on age as an independent factor in determining CPR status can lead to bias and inappropriate discrimination. One study, which focused on 6,650 patients treated at a specialist cardiothoracic centre, reported that the proportion of deaths in which DNACPR orders were in place was similar across age groups. The authors concluded that the presence of comorbidities, as measured by an organ failure score, was more influential than patient age in determining patients’ resuscitation status [26].

As we have reported above, part of the appeal to use age as a criterion is precisely the fact that it is objective and categorical. But the difficulty of then integrating age into more holistic DNACPR decisions was reflected by many of our participants, who spoke hesitantly about how age is entangled with other factors, such as comorbidities and frailty. For example, one said, “*The poor physiological reserve or the frailty that can sometimes be associated with age…but not age.”* In addition, although only two participants explicitly stated that resource stewardship might be a justification for considering age as relevant, awareness of resource scarcity is likely to be an underlying issue for many clinicians, particularly among doctors in the UK where NHS funding is such an ongoing concern. It is therefore plausible that participants who reported using age as an indicator of poor QoL and reduced rehabilitation potential were also making implicit evaluations of the opportunity cost of offering CPR for elderly patients. Even if it is unconscious or unintentional, taking into account the limitation of resources when making decisions about life-preserving interventions raises a number of ethical concerns that need to be addressed in a much more open and collective forum.

### Quality of life

The term ‘quality of life’ (QoL) appeared frequently throughout participant interviews, with two-thirds stating that it was central to their DNACPR decisions. However, the subjectivity of this term was reflected in the variation of its operationalisation. Many participants focused on baseline QoL, referring to indicators such as frailty, increasing age and dependence on others as suggestive of poor QoL prior to hospital admission. Our findings suggest that these clinicians may judge CPR to be inappropriate even if the baseline characteristics–which the patients themselves may deem acceptable– can be restored. This approach not only underscores the inherent subjectivity of the term QoL but also presents a tendency to draw indirectly on proxy observations and measures, running the risk of being influenced by personally held norms and subconscious biases. Other participants were more focused on predicting a patient’s QoL following ROSC rather than baseline characteristics. Given the complex and multifactorial nature of CPR outcomes, this also led to a significant degree of uncertainty.

Strikingly, only one-third talked about actively involving the patient when making QoL judgements; this lack of consideration of individual circumstances or possible cultural factors is particularly relevant with regard to the use of standard QOL indicators such as ‘high burden of care on families’, ‘lack of independence’ or ‘admission to care home’. These seemingly straightforward criteria can mean very different things for patients from diverse cultural backgrounds. For example, the collective responsibility of unwell family members is a core cultural value among communities from many ethnic backgrounds [27]. The strength of the relationship between functional ability and physical health has also been found to be heavily influenced by cultural factors [28]. The high-profile court judgements of Tracey v Cambridge Hospitals NHS Foundation Trust and Winspear v City Hospitals Sunderland NHS Foundation Trust highlighted that not involving patients and families in DNACPR decisions, in the absence of reasons that doing so could cause the patient significant physical or psychological harm, is likely to be in breach of Article 8 of the European Convention on Human Rights, which protects patients’ right to respect for their private and family life [29, 30].

### Frailty

The conceptualisation of frailty also varied among participants, with half focusing predominantly on making their own assessment as to whether a patient was dependent on others or had mobility difficulties. Such evaluations, which are based on brief encounters with patients and often formulated from ‘end of the bed’ impressions, are unlikely to capture the dynamic and multifactorial nature of frailty. Even for those who used the validated CFS, there was wide variation concerning the stage at which a patient’s journey was deemed most relevant. Although the use of CFS has been shown to be better at predicting mortality following CPR than drawing solely on age or comorbidities [31], the NHS Clinical Frailty Network states that clinicians must ask patients or others what the patient’s capability was two weeks ago and not base their assessment solely on the present [32]. This is because patients at the time of admission may well be impacted by an acute illness episode.

During our interviews, the overlap between assessments of QoL and frailty, even if not an issue theoretically, suggests that ideas of frailty can often extend beyond considerations of physical health. This finding is supported by a previous study that identified living situation and functional status as factors that increase the likelihood of implementing a DNACPR order [33]. Similarly, any prior inability to carry out daily living activities was closely associated with receiving a DNACPR order for patients admitted to hospital with stroke [34]. Neither of these studies explored whether the basis for taking these factors into account was to aid clinicians’ assessments of general physical health or whether they operate as proxy measures of QoL.

### Successful outcomes

The perceptions of the term ‘success’ also reflected a high degree of variation. Participant 11’s view of an unsuccessful outcome was one that resulted in the *“family wishing the patient was not alive”.* This not only illustrates the vast range of considerations implicated in the idea of ‘success’, but also how some of them may deviate from the patient’s best interests specifically, to include a focus on that of the family or healthcare provider. Although a few participants took a strictly biomedical view of success as achieving ROSC, most combined medical and functional parameters with more subjective criteria, such as QoL, the degree to which a patient was satisfied with their life, and what the clinician themselves felt was a reasonable outcome. Once again, this not only suggests a degree of ad hoc variation but might also be the potential basis for unintended and underlying bias against certain kinds of patients.

### Limitations

Reflexivity was an important consideration during the interview and analysis phases of the study. The researcher’s role as an NHS doctor meant that they assumed insider status, which may have mitigated the effect of social desirability bias, as participants may have been less concerned about damaging public confidence in clinicians when discussing the cognitive processes underlying their DNACPR decisions. While the sample size of the study was small, it is important to note that the aim of the study was to demonstrate the existence of variation in the way that DNACPR orders are implemented among clinicians, rather than offering findings that can claim statistical generalisability. Although a high degree of variability was revealed in the data, the true degree of variation is nevertheless likely to be greater than that illustrated. Further variation and inclusion of other factors is likely with a larger sample size, even if the main groupings remain the same. This is further underscored by our recruitment method, which inevitably meant that participants shared one or more characteristics (such as previous educational institutions, NHS employment trusts, or social circles). The fact that, despite this, there was much diversity in the factors raised, how they are assessed, and the degree to which they were drawn on in decision-making makes clear the extent to which this variation is likely to exist in clinical practice. While interviewing via video calls has obvious drawbacks, there is also evidence that, beyond practical advantages, participants often feel more comfortable and open to discussing sensitive and complex topics at a distance [35]. It is also important to note that this study focused exclusively on clinicians working in adult medicine and did not capture perspectives from neonatal or paediatric specialists, where different ethical and clinical considerations may apply.

The extended data collection period was primarily due to the reality that snowball sampling requires time for referral chains to develop, and then recruitment challenges during the COVID-19 pandemic when NHS staff had extremely limited availability. Clearly, those participants interviewed earlier (2021–2022), soon after the pandemic, may have recalled experiences that may have influenced their perspectives on resource allocation and DNACPR decisions. However, our thematic analysis did not reveal systematic differences in responses based on interview timing, suggesting that core decision-making processes remained relatively stable.

## Conclusion

This small qualitative study demonstrated that there may be an inevitable tension in clinical decisions regarding DNACPR orders. On the one hand, there is real strength in enabling individual practitioners to evaluate patients on a case-by-case basis and adopt an intuitive approach that allows them to arrive at an overall position on the appropriateness of a DNACPR order on the basis of multiple, and sometimes incommensurate, assessments. On the other hand, the absence of a national framework that incorporates objective measures to aid clinicians in predicting CPR outcomes not only leads to significant variation but also, potentially more importantly, permits implicit values and bias to shape assessments without the clinician or others even being aware of it.

Although clinical decision aids of all kinds are designed to facilitate standardisation of decisions on the basis of an algorithm of the current evidence and the utilisation of objective measures, qualitative studies such as this suggest there is a possible limitation to this kind of approach. While the synergistic nature of variables such as the presence of comorbidities, poor mobility, and increasing age can enable clinicians to take a more holistic approach to patient health, it is very challenging to measure and weigh each of these factors against others. This can lead to too much or too little focus on certain characteristics. For example, the use of age as an informal proxy indicator of QoL or frailty—as well as acting as an independent factor in and of itself—means that the impact of taking age into account for DNACPR decisions may be greater than clinicians are consciously aware of.

The tendency for factors to serve as proxies for one another suggests that apparent objectivity may mask subjective judgements. While enhancement of national guidelines to assist doctors in the application of quantitative measures of health may help clinicians approach DNACPR decisions in a more informed manner, and while this may mitigate the influence of unconscious biases, it is challenging to balance this against the risk of depriving doctors of the professional autonomy they draw on to make nuanced, individualised DNACPR order assessments. Recognition of this tension is fundamental to developing policies that acknowledge both the value of clinical autonomy and the need for bias mitigation.

## Supplementary Information

Below is the link to the electronic supplementary material.

Supplementary Material 1

## Data Availability

Interview transcripts have not been made publicly available in the interest of participant anonymity. However, they are available from the corresponding author upon reasonable request. The interview guide has been made available in the supplementary information.

## Abbreviations

DNACPR: Do not attempt cardiopulmonary resuscitation
CPR: Cardiopulmonary resuscitation
NHS: National health service
QoL: Quality of life
CQC: Care quality commission
BMA: British medical association
ERC: European resuscitation council
GMC: General medical council
LSHTM: London school of hygiene and tropical medicine
CFS: Clinical frailty scale
ROSC: Return of spontaneous circulation
ICU: Intensive care unit
