# Supplementary material for: Navigating DNACPR decisions: a qualitative study of DNACPR factor variability and implementation among NHS Doctors
Source: BMC Med Ethics. 2026 Jan 19;27:22. doi: 10.1186/s12910-025-01286-2 (PMC12895626; doi:10.1186/s12910-025-01286-2)
Supplement: Supplementary file 1 — Supplementary Material 1 [file 12910_2025_1286_MOESM1_ESM.pdf]

Interview Guide

Section 1- Introductory questions

- Tell me a bit about your professional role, how long you have been a clinician and in what speciality do you work?
- How involved are you with making DNACPR decisions? How often do you find yourself having to make these decisions?

Section 2 - Factors that may influence your decision-making

- What kinds of factors do you take into account when you make DNACPR decisions?
- What is your idea of a successful and unsuccessful CPR outcome?
- What patient factors do you consider when assessing the likelihood of the success of CPR?
- Exploration of reasoning behind the use of any factor mentioned by the participant. e.g If frailty, quality of life, age or comorbidities are mentioned- how and why do you assess each of these factors?
- Do you sometimes feel like these factors compete? If so, how do you navigate this?
- Do you use or know of any guidelines when making DNACPR decisions?

Section 3- Concluding questions

- Do you feel your colleagues have differing views on this?
- Do you ever need to negotiate DNACPR decisions with colleagues/patients/families and if so how do you navigate this?
- When do you feel it is appropriate to involve patients and relatives, and when do you not?
- Finally, is there anything you’d like to say on the topic that I haven’t explicitly asked you about?
